# Effects of high-order interactions on synchronization of a fractional-order neural system

**DOI:** 10.1007/s11571-023-10055-z

**Published:** 2024-01-08

**Authors:** İbrahim Ethem Saçu

**Affiliations:** Clinical Engineering Research and Implementation Center (ERKAM), Erciyes University, 38030 Kayseri, Turkey

**Keywords:** High-order relations, Fractional-order neural system, Synchronization, Second-order interactions, PSO

## Abstract

In this study, effects of high-order interactions on synchronization of the fractional-order Hindmarsh–Rose neuron models have been examined deeply. Three different network situations in which first-order coupling, high-order couplings and first-plus second-order couplings included in the neuron models, have been considered, respectively. In order to find the optimal values of the first- and high-order coupling parameters by minimizing the cost function resulted from pairwise and triple interactions, the particle swarm optimization algorithm is employed. It has been deduced from the numerical simulation results that the first-plus second-order couplings induce the synchronization with both reduced first-order coupling strength and total cost compared to the first-order coupled case solely. When the only first-order coupled case is compared with the only second-order coupled case, it is determined that the neural network with only second-order couplings involved could achieve synchronization with lower coupling strength and, as a natural result, lower cost. On the other hand, solely second- and first-plus second-order coupled networks give very similar results each other. Therefore, high-order interactions have a positive effect on the synchronization. Additionally, increasing the network size decreases the values of the both first- and high-order coupling strengths to reach synchronization. However, in this case, total cost should be kept in the mind. Decreasing the fractional order parameter causes slower synchronization due to the decreased frequency of the neural response. On the other hand, more synchronous network is possible with increasing the fractional order parameter. Thus, the neural network with higher fractional order as well as high-order coupled is a good candidate in terms of the neural synchronization.

**Supplementary Information:**

The online version contains supplementary material available at 10.1007/s11571-023-10055-z.

## Introduction

One of the most important phenomena in terms of neuron modeling is synchronization. It is considered that synchronization is a natural result of collective behaviors of neurons firing in a coordinated and rhythmic manner. Neural synchronization has been linked to several brain activities such as consciousness, attention, memory, and perception. It is assumed to be vital for the integration of information across different brain regions (Dayan and Abbott 2001). In addition, its disruption has been associated with various neurological and psychiatric disorders. Investigation of relationships between some diseases like epilepsy, Parkinson’s and synchronization has also been ongoing research (Squire et al. 2008). Therefore, understanding the mechanisms underlying neural synchronization and its modulation is of great importance in neuroscience research.

Complex network theory provides a framework for studying the structure and dynamics of interacting systems. It is expressly considered that the foundation for linkages between the system components is dyadic or pairwise interactions. However, in order to fully explain many complex systems, structural modeling of networked systems must be improved further. It is not possible for classical networks, which characterize pairwise connections, to fully capture group relationships in complex systems. This circumstance makes it difficult to describe complicated behaviors. To solve these restrictions, higher-order networks, in which linkages can connect more than two nodes, have arisen as a new area in network research. Higher-order structures, such as simplicial complexes and hypernetworks, have been proposed as useful models to explain the structure of a wide range of technical, biological, social, and other situations that are encoded in group interactions involving three or more members. Simplicial complexes are a strong mathematical basis for describing higher-order networks involving the interactions of nodes at different granularities. It broadens the notion of a graph by including higher order interactions like triangles, tetrahedra, and higher dimensional simplices (Majhi et al. 2022; Alvarez-Rodriguez et al. 2021; Bick et al. 2023; Boccaletti et al. 2023).

Due to utmost importance of the synchronization, there are lots of reports in the literature about coupling mechanism and types, synchronization control, coupling strength, topology, noise different states of the coupled networks and so on (Belykh et al. 2005; Casado 2003; Ma and Tang 2017; Ma et al. 2017; Gerstner et al. 2014; Sun et al. 2019; Usha and Subha 2019; Guo et al. 2017; Shajan et al. 2021; Xu et al. 2017; Elson et al. 1998; Li et al. 2013). However, coupling mechanism in these studies usually involves only pairwise relations, namely first-order couplings. On the other hand, some up to date studies have shown clues about that high-order relations in neural networks of the brain are available (Petri et al. 2014; Lord et al. 2016). Understanding high-order interactions in neural systems has significant implications for different fields such as neuroscience and robotics. It can help us develop more accurate models of neural activity and behavior, leading to improved diagnostic and therapeutic approaches for neurological disorders. Additionally, insights gained from studying high-order interactions in neural systems can inform the development of more advanced machine learning as well as artificial intelligence algorithms. High-order interactions/correlations, also known as multi-way interactions refer to the simultaneous effects of three or more variables on a system/process. In neural systems, the high-order relations are called “simplicial complexes” which construct body interactions among neurons of network. Higher order couplings, thus, encompass not just pairwise linkages but also triple, quadruple, and other interactions. For instance, a 0-simplex corresponds to a single neuron, a 1-simplex means a line segment connecting two neurons, a 2-simplex is a triangle formed by connecting three neurons, and so on (Gambuzza et al. 2021; Battiston et al. 2020). The interactions among *m* + 1 nodes can be presented by *m* simplex in the simplicial complexes. Kuramoto oscillators constructed in the 2-simplex have been studied in Millan et al. (2020) and it is shown that there is explosive synchronization. Another study results that high-order interactions cause the rapid synchronization in the high-order phase oscillators in Skardal and Arenas (2021). It is observed in Gallo et al. (2022) that the application of directed higher-order interactions might disrupt the synchronization state in a higher-order network consisting of eight Rössler oscillators. The synchronization in a multiplex network within each layer, the Rössler oscillators coupled through diffusive pairwise and non-pairwise connections have been examined in Anwar and Ghosh (2022) and it is concluded that higher-order interactions improve intra-layer synchronization and increase the robustness of interlayer synchronization in comparison to the pure pairwise situation.

High-order interactions have been involved in a neural network consisted of the Morris-Lecar (ML) neuronal models in Tlaie et al. (2019) and it is reported that time ordered synchronization and fast information transfer are possible by means of high-order interactions. It is stated in Ince et al. (2009) that neural dynamics in rat somatosensory cortex can not modelled in detail by employing only first-order interactions. Additionally, the reaction of the neuro-information propagation to high-order interactions has also been investigated in that study. Existence of the high-order interaction in neuronal activity has been shown in Amari et al. (2003). The effects of the three body interactions along with pairwise interactions on the synchronization of the Hindmarsh–Rose (HR) neurons have been evaluated in Parastesh et al. (2022) and it is concluded that overall synchronization can be achieved with reduced cost and lower first-order coupling parameters in presence of the second-order interactions. High-order interactions and electromagnetic induction together have been included in HR and ML neuron models in Ramasamy et al. (2022) and it is mentioned that even though flux coupling leads to increased coupling strength parameters for synchronization of HR neurons, it makes synchronization possible with lesser strength parameters in a network of ML neuron models. The synchronization of a higher-order network of the memristive Rulkov model with several kinds of synapses has been explored in Mehrabbeik et al. (2023a) and it is shown that higher-order interactions alter synchronization patterns to lower multi-node chemical coupling parameter values. The impact of higher-order chemical interactions and pairwise connections on the synchronization of a network comprising globally coupled Rulkov neuronal models has been investigated in Mirzaei et al. (2022). The findings demonstrate that when more neurons are engaged in the network, multi-node interactions can significantly improve neural network synchronization more so than pairwise interactions. The influence of pairwise and non-pairwise interactions on the synchronization coupled memristive HR neuron maps has been addressed in Mehrabbeik et al. (2023b). The findings indicate that synchronization among the neurons is achieved through chemical pairwise and non-pairwise synapses, even with the presence of the weakest coupling strengths.

On the other hand, the aforementioned studies are primarily based on integer-order mathematics. However, a more general form of this classical approach can be achieved through fractional calculus, enabling more accurate and wide-ranging modeling. As a result, fractional calculus has diverse applications across different areas such as engineering, biology, medicine, and many more (Baleanu et al. 2016; Sun et al. 2018; Dalir and Bashour 2010; Podlubny 1998). Unlike classical neuron models, fractional neuron models offer various opportunities such as the possibility to treat the fractional order as a separate bifurcation parameter and thus achievement of new rich dynamics, the memory effect due to the dependence on entire past values inherent in fractional derivative definitions, power law dynamics, spike time adaptation and the ability to define coupling functions in a fractional manner (Ionescu et al. 2017; Drapaca 2017; Lundstrom et al. 2008; Teka et al. 2018; Korkmaz and Saçu 2022). Memory effect of the fractional neuronal models are supported by representation of the cell membrane with the coupled active conductances. Neural dynamics with multiple time scales can be interpreted via the fractional calculus. Significant studies concerning fractional neuron models, their dynamics, synchronization and synchronization control have been extensively addressed in the literature (Liu et al. 2021; Ding et al. 2021; Malik and Mir 2022, 2021, 2020; Xie et al. 2014; Kaslik and Radulescu 2017; Song and Cao 2014; Tolba et al. 2019; Upadhyay and Mondal 2015; Shi and Wang 2014; Dar et al. 2021; Yang et al. 2021; Giresse et al. 2019; Meng et al. 2020; Jun et al. 2014). On the other hand, the fact that fractional neural networks support infinitive memory and inherited features distinguish them from classical neural networks. In addition, the data processing, detection of stimulations and anticipation capabilities of fractional neural networks are getting better (Song and Cao 2014). It is expected that fractional recurrent neural networks can provide better parameter estimation. The rate of network approximation increases with including fractional calculus into the network (Song and Cao 2014). Rich dynamics such as bifurcation, chaos and multi-stability can be achieved with fractional neural networks. Again, it can be said that fractional-order neural networks are effective in system identification (Kaslik and Radulescu 2017). However, beyond all these comprehensive studies, the influence of high-order interactions on the synchronization characteristics of fractional neuron models has not yet been adequately addressed. With this motivation, in this study, the impacts of high-order interactions on neural synchronization in a neural network consisting of fractional-order HR neurons have been discussed and the following points are sought to be clarified:

(i) Determining whether high-order interactions have a positive or negative effect on neural synchronization in the neural network composed of fractional-order neuron models,
(ii) To achieve synchronization, determination of the most appropriate first- and high-order coupling strengths by considering the cost function,
(iii) Impact of fractional order parameter on neuronal synchronization
(iv) Impact of network size on neural synchronization

In this context, the fractional-order HR neuronal model and preliminaries have been introduced in section"preliminaries and fractional-order hr neuronal model". The first- and second-order couplings of the fractional neuron models and the obtained simulation results have been presented in section"Coupling of the fractional-order neuronal models". In section"Coupling of the fractional-order neuronal models", the determination of the optimal first- and second-order coupling parameters is also presented, taking into account the cost function. The effects of network size and fractional order parameter on neural synchronization have been examined in sections "Effect of the fractional order of the neural network" and "Effect of the size of the neural network", respectively. The evaluation of the results and future studies are provided in section "Conclusion".

## Preliminaries and fractional-order HR neuronal model

When long-term interactions, dynamic processes, and the continuous flow of time are taken into account in the analysis and modeling of physical or natural phenomena, the fractional integral emerges as a key instrument. From this perspective, the Riemann–Liouville fractional integral is one of the most widely recognized and extensively utilized definitions of fractional integrals, while there are several definitions and forms that may be found in the literature.

The fractional integral definition of Riemann–Liouville type with order *v* is given as below (Baleanu et al. 2016)

1 $$ {}^{RL}J^{v} g(t) = \frac{1}{\Gamma (v)}\int\limits_{0}^{t} {\frac{g(s)ds}{{(t - s)^{1 - v} }}} $$

where *v* ∈ (0, 1), *g*(*t*) is a continuous function and Γ(.) is Gamma function. On the other hand, the fractional derivative definition of Caputo type is expressed as following (Podlubny 1998)

2 $$ {}^{C}D^{v} g(t) = \frac{1}{\Gamma (1 - v)}\int\limits_{0}^{t} {\frac{{g^{\prime}(s)ds}}{{(t - s)^{v} }}} $$

where *g'*(*s*) is first order derivative of the function *g*(*s*). With the presence of integer order derivatives in the initial conditions, Caputo type fractional derivatives are simpler to interpret. Additionally, a constant's derivative under the Caputo definition is zero as is usual in integer calculus. A general type of fractional differential equation with order *v* is given as following (Podlubny 1998)

3 $$ \begin{aligned} D^{v} g(t) &= f(t,g(t)) \hfill \\ g(t_{0} ) &= g_{0} \hfill \\ \end{aligned} $$

where *D*^*v*^ is fractional derivative operator of Caputo type and *g*_0_ is initial condition.

To mimic the dynamics of real neurons, various neuron models have been available in the literature. Conductance-based neuron models are the type that resembles biological neurons the most. In these models, ion channels are imitated by voltage-dependent conductances, as well as the cell membrane by a capacitor. The most common model from this category is the Hodgkin-Huxley (HH) neuron model (Hodgkin and Huxley 1952), which allows for rich dynamics to be obtained. However, this model comes with significant computational and hardware implementation costs. Especially if a network structure to be created using these models is considered, the cost becomes even higher. On the other hand, different neuronal models have been proposed to overcome these limitations. One of them is the FitzHugh-Nagumo (FHN) neuronal model, which is represented by 2 dynamic variables (Fitzhugh 1969). This model is important in terms of showing the spiking behavior of a real neuron. However, the dynamic behaviors that can be obtained with this model are very limited. The 3-variable Hindmarsh–Rose (HR) neuronal model is derived by adding another dynamic variable to the FHN model (Hindmarsh and Rose 1984). Thus, it becomes biologically more realistic and captures several important features of neural dynamics. The HR neuronal model is salient in terms of being low-cost and providing richer dynamic diversity. One of the indispensable features of the HR model is its ability to generate a variety of spiking patterns, involving bursting, regular spiking, and chaotic spiking. The HR model has been employed extensively in the study of synchronization in neuronal systems, both in simulations and in experiments. For example, it has been used to model the synchronization of neurons in the thalamus, and the hippocampus (McCulloch and Pitts 1943). It should also be noted that, in addition to the neuron models mentioned above, there are also different neuronal models like Integrate-and-Fire (McCulloch and Pitts 1943), Izhikevich (Izhikevich 2003), etc.

The fractional HR neural model commensurate order of *α* is defined by the following equation set (Malik and Mir 2020).

4 $$ \begin{aligned} D^{\alpha } x &= y - x^{3} + b_{h} x^{2} + I_{exs} - z \hfill \\ D^{\alpha } y &= 1 - 5x^{2} - y \hfill \\ D^{\alpha } z &= r\left( {s(x - x_{Rs} ) - z} \right) \hfill \\ \end{aligned} $$

where *α* (0 < *α* < 1) is fractional order, and the state variables *x*, *y*, and *z* correspond to respectively membrane potential, spiking variable as well as the adaptation variable. The other control parameters are set to *b*_*h*_ = 3, *r* = 0.009, *s* = 4 and *x*_*Rs*_ = −1.6 for the numerical simulations (Malik and Mir 2020). To check stability of the fractional-order system defined in (4), the minimum fractional order *α*_*min*_ should be determined according to the condition ∀*λ*_*i*_ > *α*_*min*_π/2 where *λ*_*i*_ (*i* = 1, 2, …) is the eigenvalue of the system at equilibrium points (Tavazoei and Haeri 2009). For the stimulus *I*_*exs*_ = 2.2 and parameter set given above, the minimum fractional order *α*_*min*_ is calculated as 0.5454. The predict and correct (PC) method has been utilized to numerically analyze the fractional-order system given by (4). The application procedure of the PC technique to fractional-order differential equations is given elaborately in Garrappa (2010); Diethelm et al. (2004).

**Fig. 1:**
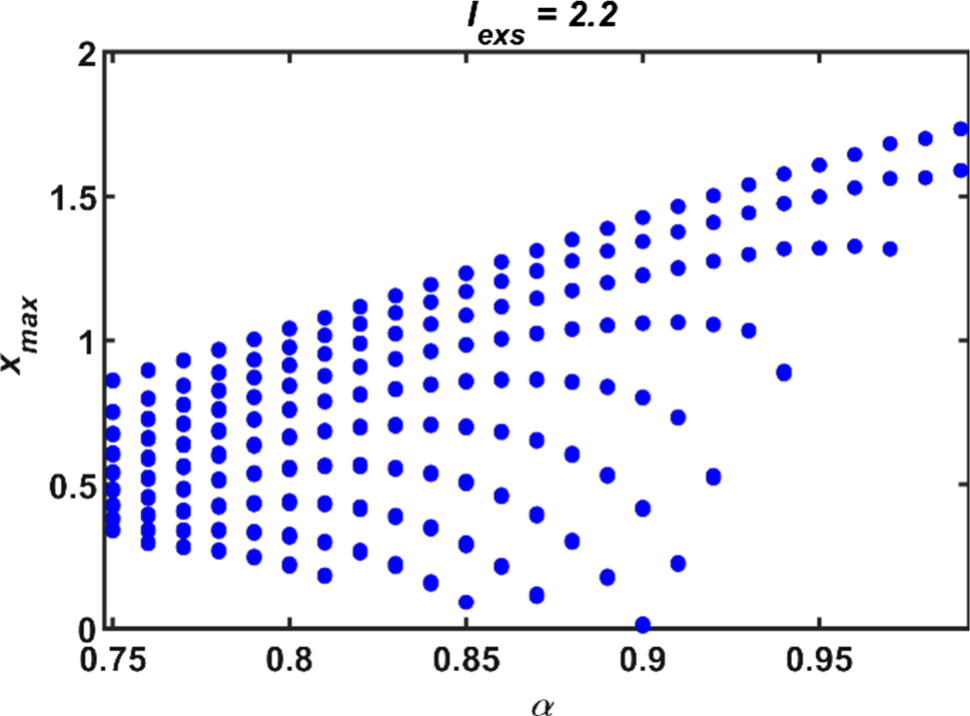
The bifurcation diagram for the fractional order *α* versus peak values of the membrane potential *x*

While model parameters are fixed to values given above except the order *α*, the bifurcation diagram of the HR neuronal model for the fractional order *α* versus *x*_*max*_, the maximum values of the variable *x*, is demonstrated in Fig. 1. It is obviously seen from Fig. 1 that pattern type of the model response has changed by means of fractional order parameter. For example, the neural model exhibits period-3 bursting for *α* = 0.95 and period-7 bursting for *α* = 0.9.

## Coupling of the fractional-order neuronal models

Electrical and chemical coupling are two pivotal mechanisms which play a critical role in the synchronization of neurons in the brain. Electrical coupling is a direct cell-to-cell communication mechanism that allows ions to flow between neurons through gap junctions. This enables for rapid and reliable synchronization between neurons, particularly in oscillatory networks. On the other hand, chemical coupling involves the release of neurotransmitters that adhere to receptors on neighboring neurons, triggering a cascade of biochemical events that eventually lead to the synchronization of the neurons. Chemical coupling is a more intricate and slower mechanism compared to electrical coupling (Usha and Subha 2019; Li et al. 2013). Various mathematical models of electrical and chemical coupling that aim to elucidate the synchronization of neurons have been developed. These models vary from simple linear models to highly complex nonlinear models, and they have been used to investigate various aspects of neural synchronization, such as the role of coupling mechanism and strength, frequency, the impact of noise and heterogeneity, and the occurrence of synchronization in large-scale networks (Gerstner et al. 2014; Guo et al. 2017). On the other hand, recent years have witnessed an emergence of studies that focus on the reliance of information transmission among neurons on the equilibrium of energy. These studies claim that when neurons are in an energy-balanced state, synaptic channels become obstructed, whereas information transfer is possible in instances of energy imbalance (Yao et al. 2023). A further step of this approach involves altering the coupling intensity of the neuron via self-adjustment, thereby attempting to achieve equilibrium in energy and synchronization (Xie et al. 2022). A disruption in energy balance can be initiated within the neuron as a result of physical stimulus, including but not limited to temperature, illumination, voice, and mechanical forces (Sun et al. 2023).

A simplicial complex order of *C*_*d*_ includes not only the first-order couplings but also high-order couplings. However, here, it is assumed as *C*_*d*_ = 2 which means only first- and second-order couplings considered. The definitive equations of a simplicial complex, that consists of fractional-order HR neuron models, is given in (5).

5 $$ \dot{X}_{ni} = F(X_{ni} ) + \sigma_{c1} \sum\limits_{j = 1}^{N} {b_{ij} g_{c1} (X_{ni} ,X_{nj} )} + \sigma_{c2} \sum\limits_{j = 1}^{N} {\sum\limits_{k = 1}^{N} {b_{ijk} g_{c2} (X_{ni} ,X_{nj} ,X_{nk} )} } $$

where *X*_*ni*_ ∈ ℝ^*s*^ corresponds to the state variables with *s* dimensional of the system, *F*(*X*_*ni*_) is the function which determines relationships among nodes, (*σ*_*c*1_, *σ*_*c*2_) are strength parameters of first- and second-order couplings. The functions *g*_*c*1_(*X*_*ni*_, *X*_*nj*_) and *g*_*c*2_(*X*_*ni*_, *X*_*nj*_, *X*_*nk*_) are respectively first- as well as second-order coupling functions. The parameter *b*_*ij*_ is a link indicator between nodes *i* and *j*, if *b*_*ij*_ equals to 1, there is a link, otherwise not. Similarly, if *b*_*ijk*_ equals to 1, there is a triangle constructed by the nodes *i*,* j* and *k*, otherwise not.

**Fig. 2:**
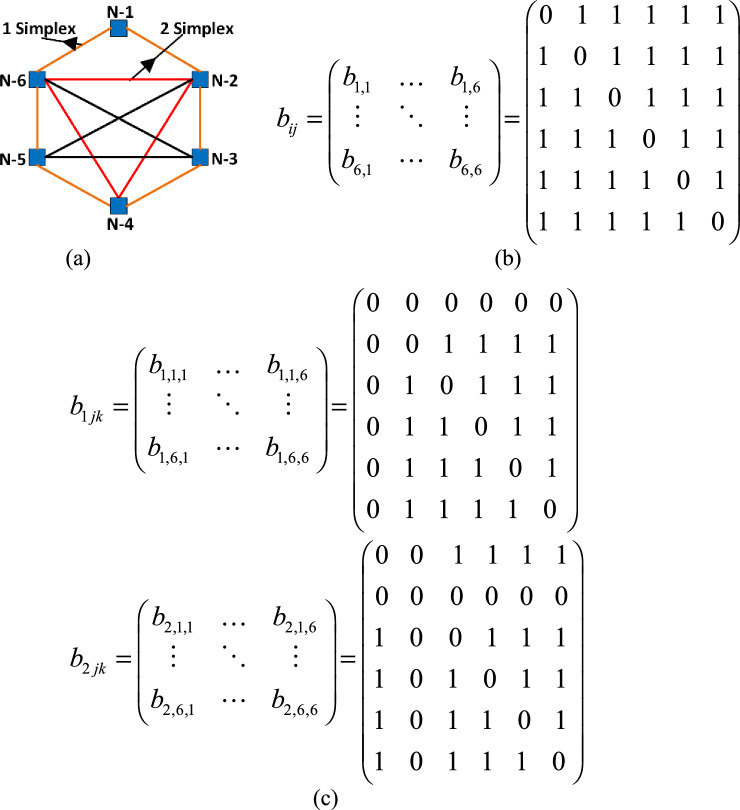
(a) Global coupling of the neural network consisted of HR neuronal models (b) first-order coupling parameters (c) second-order coupling parameters

To gain more insights about network topology, a globally coupled neural network involving six neurons (*N*_*n*_ = 6) is shown in Fig. 2a, and the parameters *b*_*ij*_ and *b*_*ijk*_ for the connections in the neural network are given in Fig. 2b and 2c, respectively.

The synchronization of the neural network constructed of the *N*_*n*_ = 10 neuronal models has been investigated. So as to evaluate the synchronization status in terms of the quantitative measures, the standard deviation results (Zhang et al. 2015), averaged error, and averaged similarity (Fan and Wang 2017) are calculated as following.

6 $$ \begin{aligned} E_{eh}& = \left\langle {\frac{1}{{N_{n} - 1}}\sum\limits_{j = 2}^{{N_{n} }} {\left\| {x_{1} (n) - x_{j} (n)} \right\|} } \right\rangle \hfill \\ \sigma_{eh} &= \left\langle {\sqrt {\frac{{(1/N_{n} )\sum\limits_{j = 1}^{{N_{n} }} {x_{j}^{2} (n) - \left[ {(1/N_{n} )\sum\limits_{j = 1}^{{N_{n} }} {x_{j} (n)} } \right]^{2} } }}{{N_{n} - 1}}} } \right\rangle \hfill \\ S_{sml} &= \frac{1}{{N_{n} - 1}}\sum\limits_{j = 2}^{{N_{n} }} {\sqrt {\frac{{ < (z_{1} (n) - z_{j} (n))^{2} > }}{{\sqrt { < z_{1} (n)^{2} > < z_{j} (n)^{2} > } }}} } \hfill \\ \end{aligned} $$

where *σ*_*eh*_, *E*_*eh*_ and *S*_*sml*_ are the standard deviation results, averaged error, and averaged similarity, respectively. *x*_*j*_(*n*) corresponds to the *n*th entity in the time series of the *j*th neuron, *N*_*n*_ is number of coupled neurons. These three synchronization performance measures are equal to zero in ideal perfect synchronization, in this way, as the dynamics of each of the neurons forming the network are coherent with each other, all three performance measures approach to zero level.

**Fig. 3:**
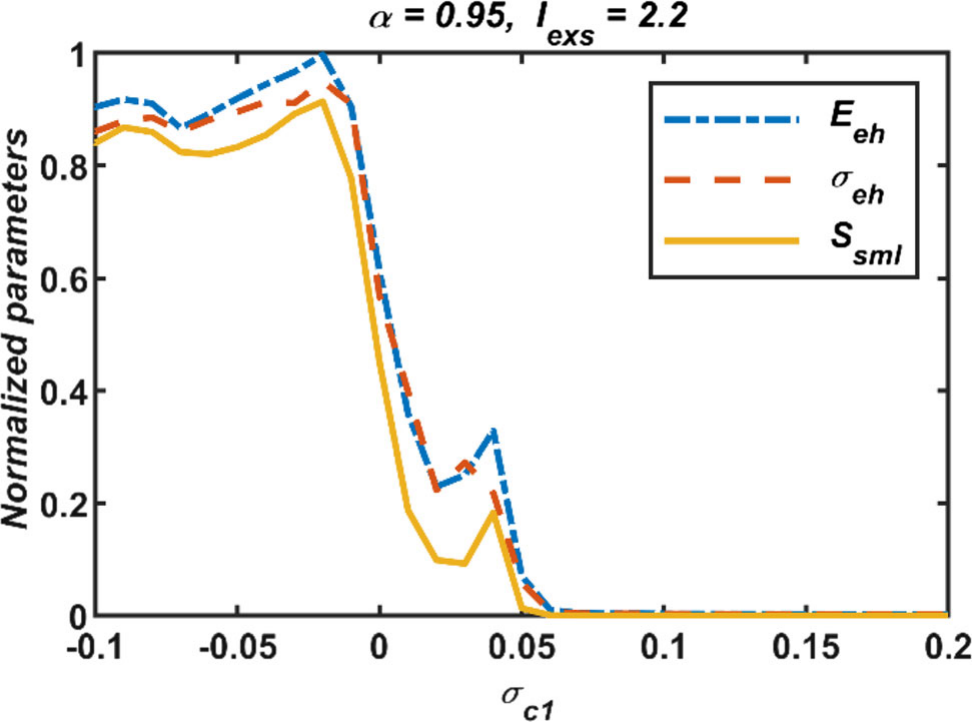
Effect of the first-order interactions on the network synchronization when the second-order interactions are passive,* σ*_*c*2_ = 0; *σ*_*c*1_: first-order coupling strength, normalized measures (*E*_*eh*_: error, *σ*_*eh*_: standard deviations, and *S*_*sml*_: similarity)

**Fig. 4:**
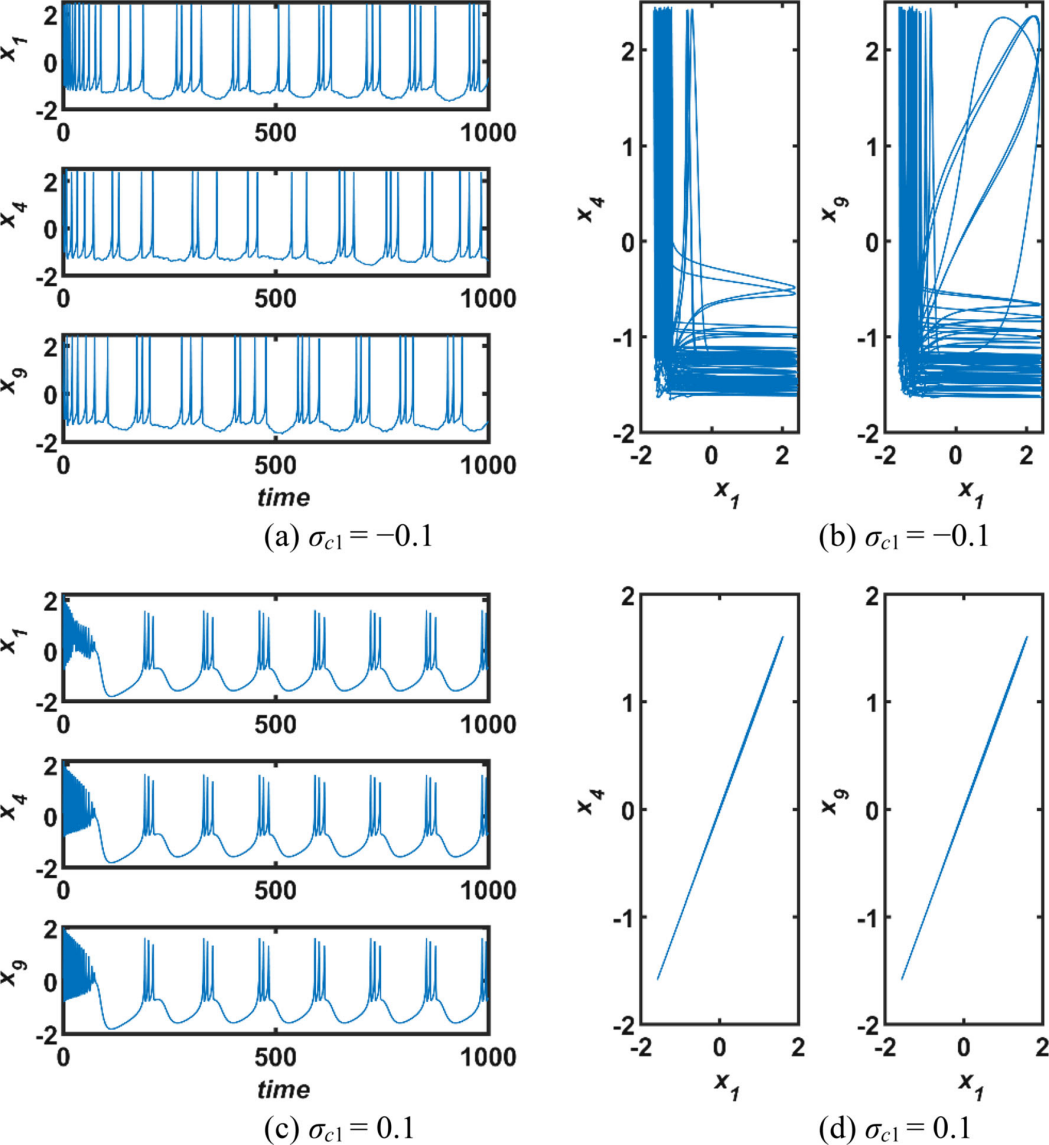
Dynamics responses of neurons in the network where first-order interactions are active only; (a,c) The temporal responses, and (b,d) phase graphs of state variables of the network; *σ*_*c*1_: first-order coupling strength

Firstly, only first-order interactions, namely, diffusive electrical couplings are considered when second order interactions are passive, *σ*_*c*2_ = 0. And thus, the synchronization status of the neural network for different values of the coupling parameter *σ*_*c*1_ is evaluated at (*α* = 0.95, *I*_*exs*_ = 2.2). The first-order coupling function is defined as *g*_*c*1_(*X*_*ni*_, *X*_*nj*_) = *X*_*nj*_ − *X*_*ni*_ (Parastesh et al. 2022). The numerical simulation results for the coupling strength *σ*_*c*1_ ∈ [− 0.1, 0.2] versus normalized performance measures are displayed in Fig. 3. It is undeniable from the figure that all three performance measures are far from zero level for the negative values of the coupling parameter *σ*_*c*1_, so the synchronization of the network is not possible in this range. However, the plots of the performance measures show a rapid decline around *σ*_*c*1_ ∈ (0, 0.05) and converge to zero with decreasing slope at positive values of parameter *σ*_*c*1_. Therefore, it can be said that the dynamic responses of the neurons in the network are compatible with each other and synchronization is possible only at positive values of *σ*_*c*1_. To better perceive this situation, the time domain responses of first, fourth, and ninth neurons in the network obtained for the strength parameter *σ*_*c*1_ = −0.1 and 0.1 are portrayed in Fig. 4. It is evidentiary from Fig. 4 that neural dynamics are synchronous for *σ*_*c*1_ = 0.1. On the other hand, it may be claimed that the standard deviation results and the averaged error values exhibit a more similar characteristic.

**Fig. 5:**
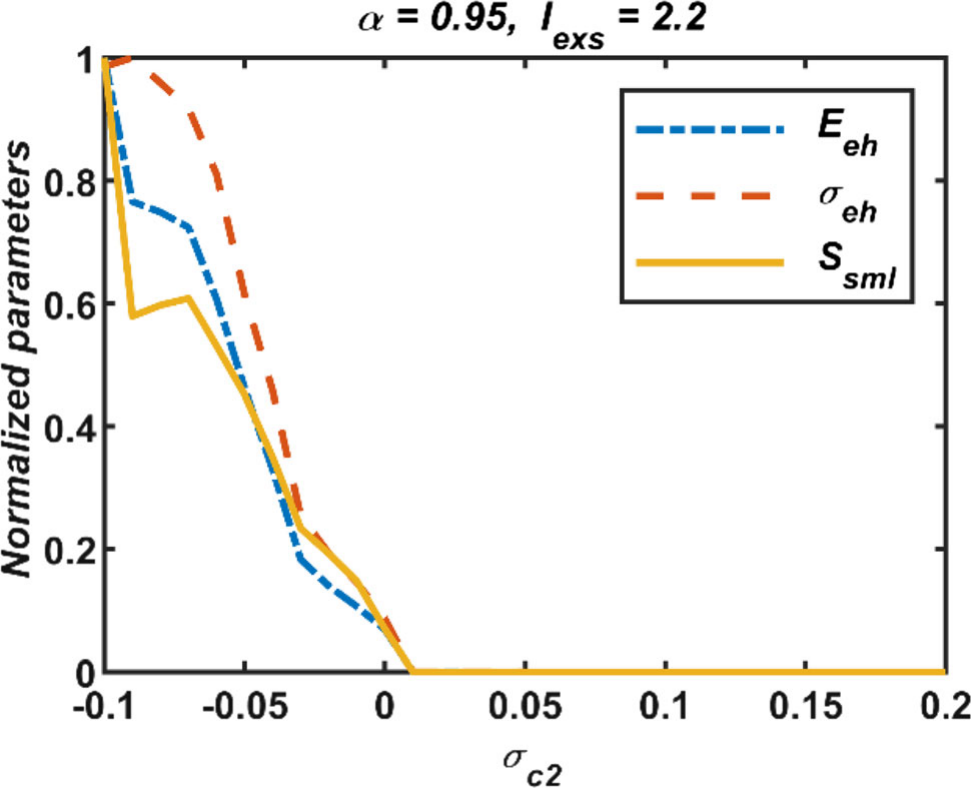
Effect of the second-order interactions on the network synchronization when the first-order coupling strength *σ*_*c*1_ is *σ*_*c*1_ = 0; *σ*_*c*2_: second-order coupling strength, (*E*_*eh*_: error, *σ*_*eh*_: standard deviations, and *S*_*sml*_: similarity)

**Fig. 6:**
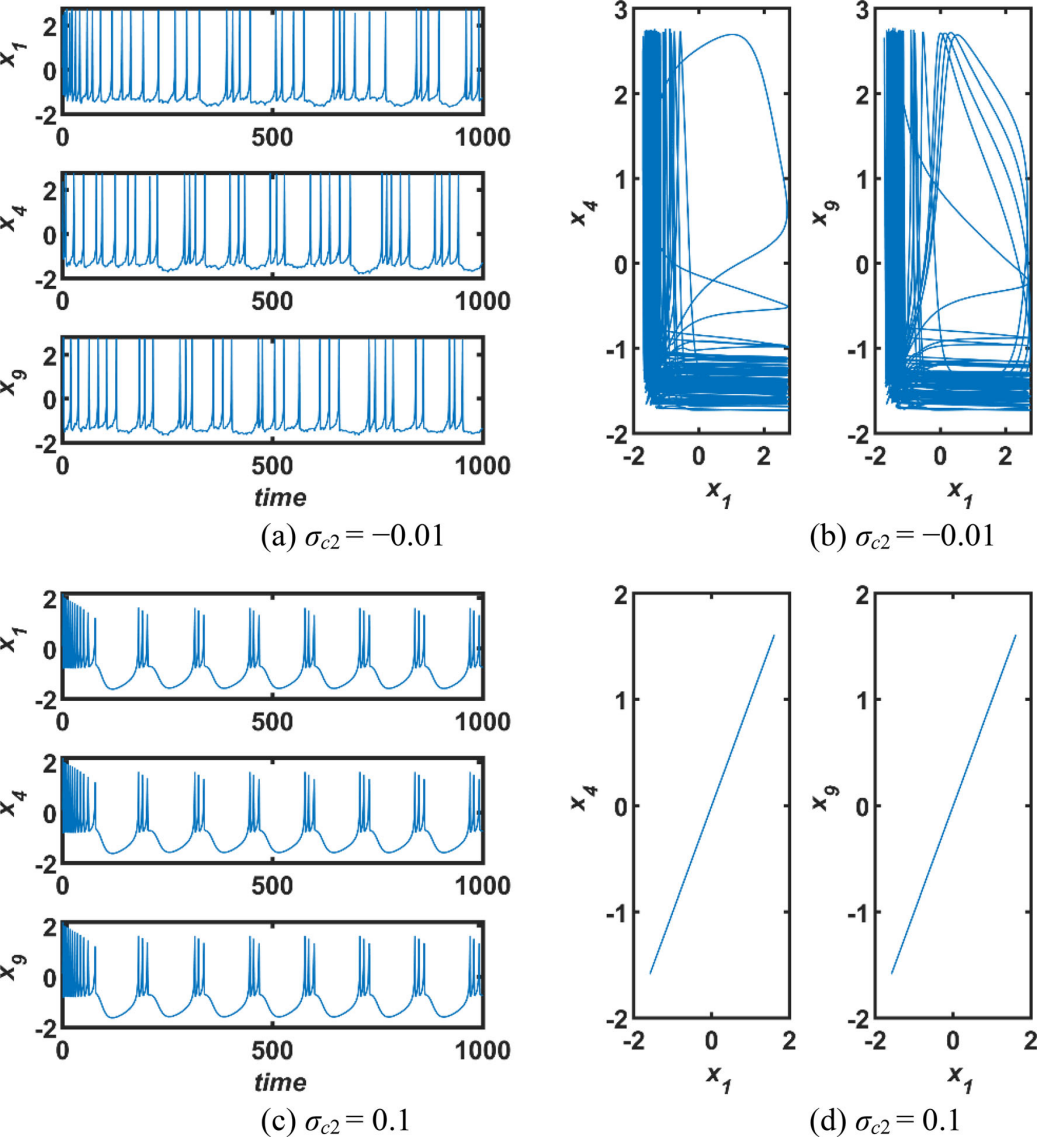
Dynamics behaviours of neurons in the network for only second-order coupling case; (a,c) The temporal responses, and (b,d) phase graphs of state variables of the network; *σ*_*c*1_: first-order coupling strength, *σ*_*c*2_: second-order coupling strength

In the second step, second-order interactions are active solely, (*σ*_*c*2_ ≠ 0, *σ*_*c*1_ = 0). In this case, the second-order coupling function is defined as *g*_*c*2_(*X*_*ni*_, *X*_*nj*_, *X*_*nk*_) = *X*_*nj*_ + *X*_*nk*_ − 2*X*_*ni*_ (Parastesh et al. 2022). The obtained simulation results for the case of (*α* = 0.95, *I*_*exs*_ = 2.2) are presented in Fig. 5. If the coupling parameter *σ*_*c*2_ is in the range (− 0.1, 0), neural synchronization is not possible owing to fact that all three performance measures are far from zero level. On the other hand, all three performance measures show a rapid decrease around *σ*_*c*2_ ∈ (0, 0.01) and converge to zero in the range *σ*_*c*2_ > 0. Consequently, the condition *σ*_*c*2_ > 0 must be met for neural synchronization to be possible. This can be observed more easily from the time domain responses and phase portraits of first, fourth, and ninth neurons in the network obtained for *σ*_*c*2_ =  − 0.01 and 0.1 in Fig. 6.

**Fig. 7:**
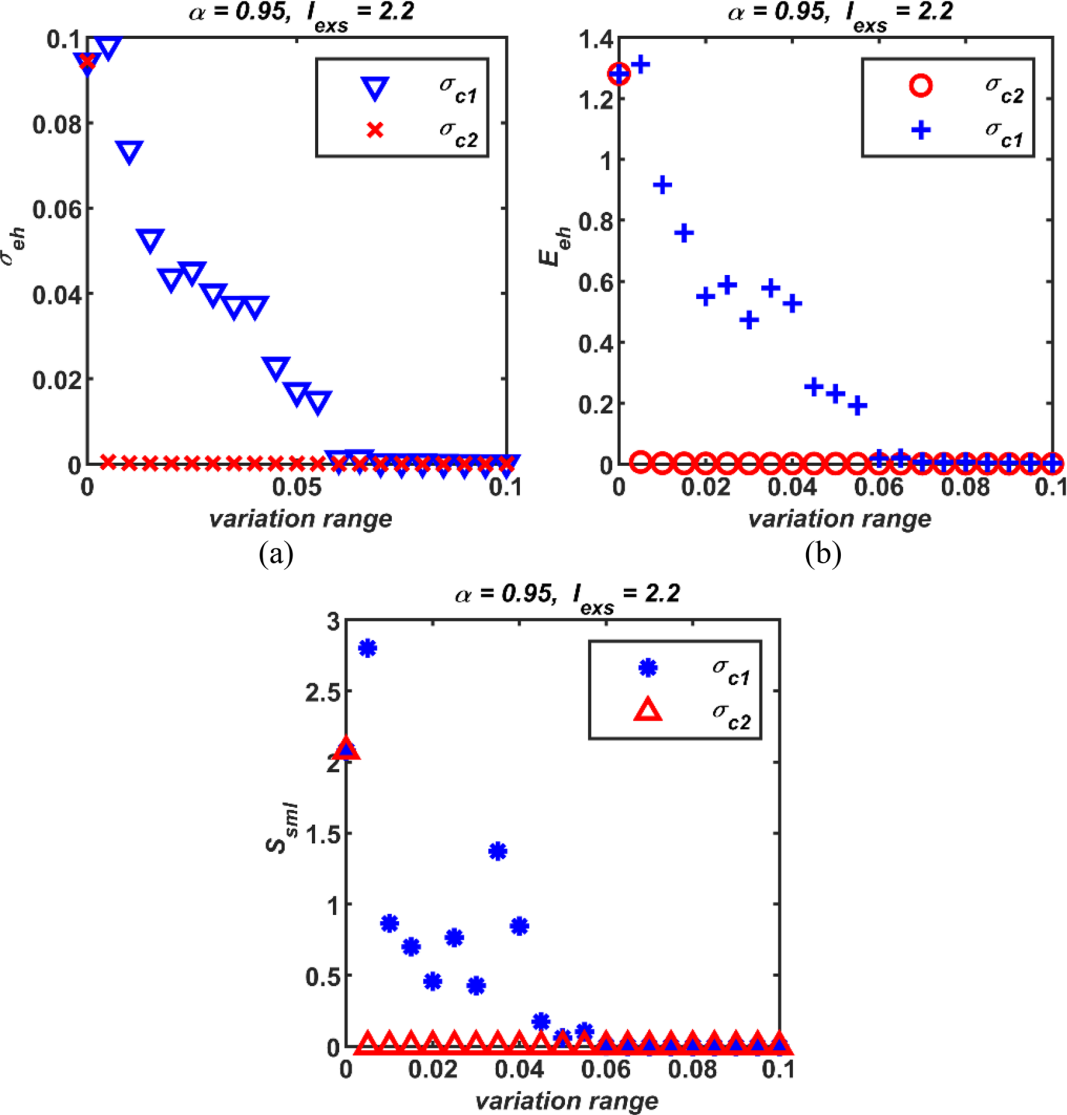
The comparison of the magnitude of the first- and second-order coupling strengths in terms of the performance measures; (a) (*σ*_*c*1_,* σ*_*c*2_) vs *σ*_*eh*_, (b) (*σ*_*c*1_,* σ*_*c*2_) vs *E*_*eh*_, and (c) (*σ*_*c*1_,* σ*_*c*2_) vs *S*_*sml*_; *σ*_*c*1_: first-order coupling strength, *σ*_*c*2_: second-order coupling strength, *σ*_*eh*_: standard deviations, *E*_*eh*_: error, *S*_*sml*_: similarity

It is distinguishable from the above simulation results that synchronization can occur in the positive values of the first- and high-order couplings. However, the magnitudes of the first- and high-order interactions required to achieve synchronization have not been compared. Therefore, at this point, one of the coupling parameters is fixed at zero while the other is changed in the range of [0, 0.1]. The obtained simulation results are presented in Fig. 7. It is noticeable from the Fig. 7 that high-order couplings produce smaller error values in terms of all three performance metrics. In other words, a much smaller *σ*_*c*2_ than *σ*_*c*1_ is demanded to attain the same synchronization performance. Thus, in the case of *σ*_*c*1_ = *σ*_*c*2_, it can be clearly said that only the second-order coupled network is more synchronous than the solely first-order coupled network.

**Fig. 8:**
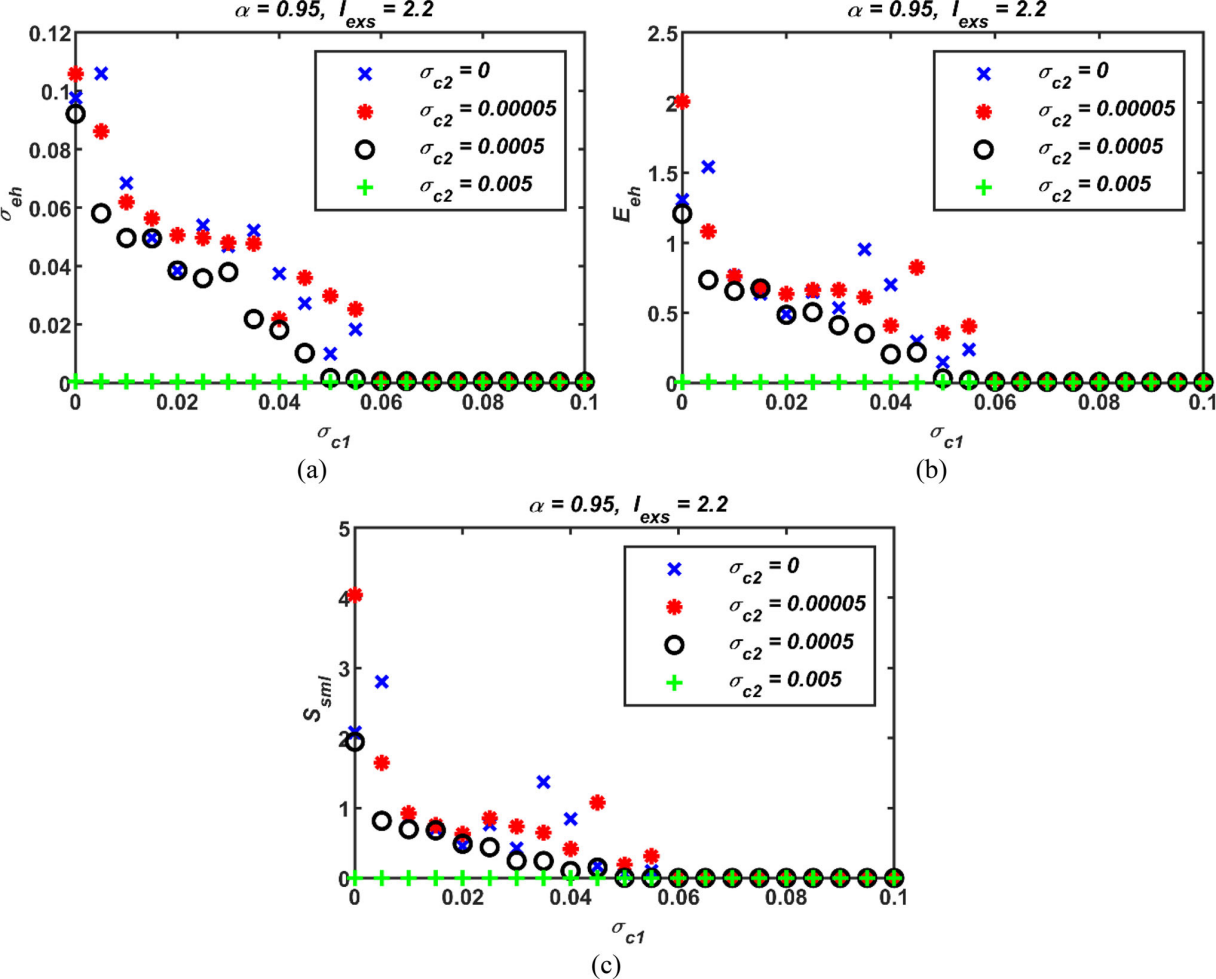
Three performance measures for different first- and second-order coupling strengths active together; (a) *σ*_*c*1_ vs *σ*_*eh*_ for several values of *σ*_*c*2_, (b) *σ*_*c*1_ vs *E*_*eh*_ for different values of *σ*_*c*2_, and (c) *σ*_*c*1_ vs *S*_*sml*_ for different values of *σ*_*c*2_; *σ*_*c*1_: first-order coupling strength, *σ*_*c*2_: second-order coupling strength, *σ*_*eh*_: standard deviations, *E*_*eh*_: error, *S*_*sml*_: similarity

Fourthly, while the coupling parameter *σ*_*c*1_ is spanned from 0 to 0.1, the numerical simulation results carried out for several values of the high-order coupling parameter *σ*_*c*2_ are demonstrated in Fig. 8. As can be clear from Fig. 8 that as the value of *σ*_c2_ increases, all three performance metrics reaches smaller errors at smaller values of *σ*_*c*1_. That is, smaller values of *σ*_c1_ are enough to achieve synchronization in the presence of *σ*_*c*2_ compared to the case where *σ*_*c*2_ is passive. It can even be said that synchronization can be achieved regardless of the value of *σ*_*c*1_ when the value of *σ*_*c*2_ exceeds a certain threshold. Moreover, it is clearly evident from the simulation results that all three performance metrics yield parallel results. Therefore, from this point on, only the standard deviation results have been considered as the performance metric in the evaluations.

**Fig. 9:**
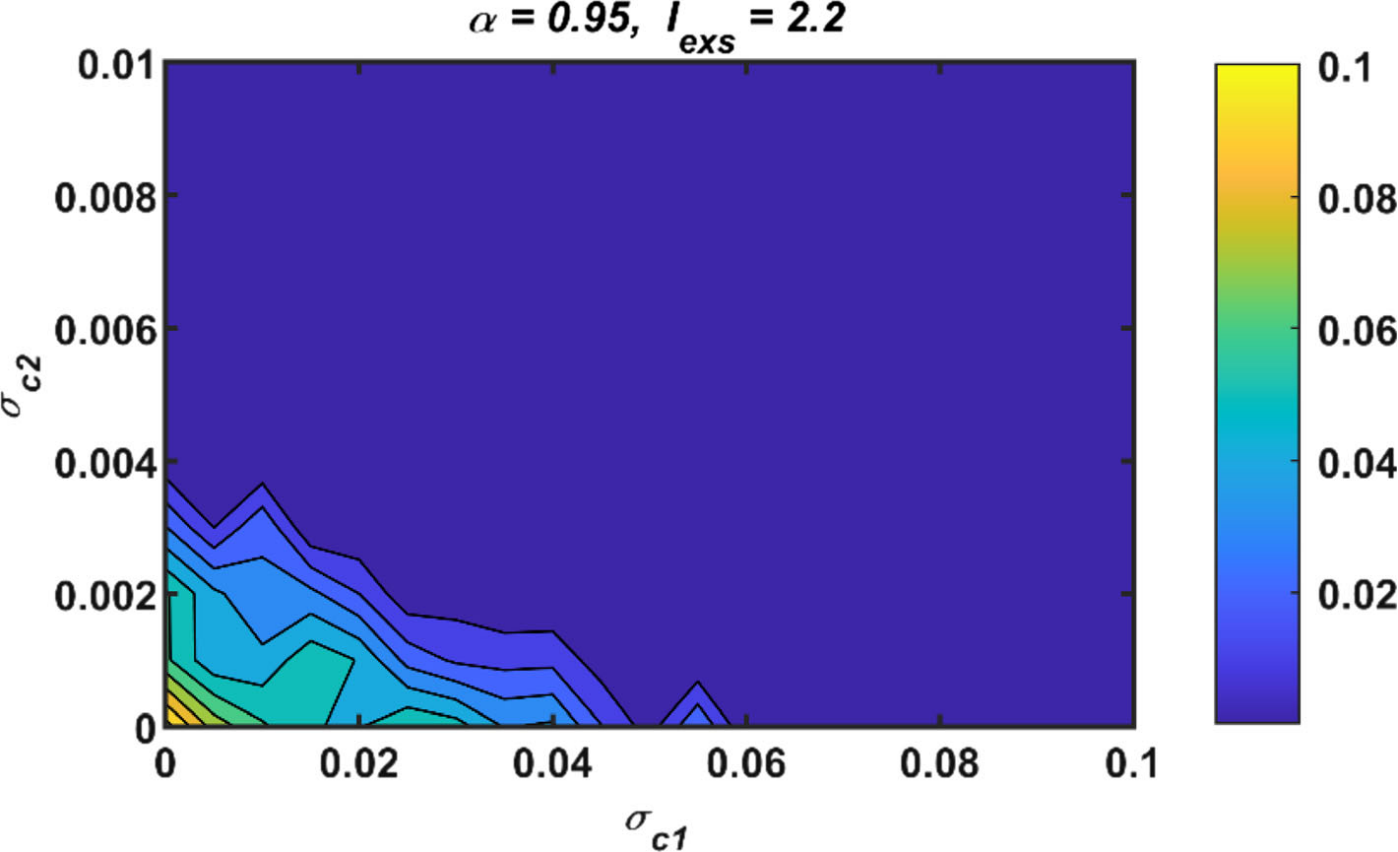
Contour plot of *σ*_*c*1_ vs *σ*_*c*2_ for the standard deviation results *σ*_*eh*_;* σ*_*c*1_: first-order coupling strength, *σ*_*c*2_: second-order coupling strength

The contour plot of first-order (*σ*_*c*1_) vs second-order (*σ*_*c*2_) interactions based on the calculated standard deviation results is portrayed in Fig. 9. The shapes of contours show that there is not a perfect linear relationship. So, optimization algorithms can be utilized to determine the most optimal coupling parameters that keep the performance metric below a certain threshold. Optimization algorithms are widely used in broad fields such as engineering, biology, medicine, and computer science.

On the other hand, although the presence of *σ*_*c*2_ reduces the value of *σ*_*c*1_ required for synchronization, a more accurate decision can be made if the effectiveness of this situation is evaluated considering the cost. In this context, the cost function can be defined as in (7) (Parastesh et al. 2022).

7 $$ C_{{{\text{c}} st}} = \sigma_{c1} \frac{{N_{n} (N_{n} - 1)}}{2} + \sigma_{c2} 3\frac{{N_{n} (N_{n} - 1)(N_{n} - 2)}}{6} $$

Here, *N*_*n*_ is the number of neurons, *N*_*n*_(*N*_*n*_ − 1)/2 is the links formed by the pairwise connections between neurons, and the expression 3*N*_*n*_(*N*_*n*_ − 1)(*N*_*n*_ − 2)/6 is the triangles formed by the triple connections among the neurons.

There are many optimization algorithms available in literature, each with its own advantages and disadvantages. Several of the most commonly used optimization algorithms include gradient descent, genetic algorithms, particle swarm optimization, and ant colony optimization (Gad 2022). In this study, Particle Swarm Optimization Algorithm (PSO) has been exploited to determine the optimum first- and high-order coupling parameters that will minimize the cost function given by (7). (PSO) is a metaheuristic optimization algorithm, that is inspired by the social behavior of bird flocks or fish schools (Gad 2022). In PSO, a group of particles represents the possible solutions to the problem of interest, and each particle adjusts its position in the search space based on its own experience and that of its neighbors. The particles move towards the best-known position in the search space, which is updated as new, better positions are discovered. PSO is characterized by its simplicity, efficiency, and powerful implementation. It has been successfully applied to a broad range of optimization problems, including engineering design, data mining, and machine learning. Here, while the performance metric *σ*_*eh*_ is below a certain limit value, the cost function given by (7) is tried to be minimized by searching for the best candidate in the solution space. Thus, coupling parameters are determined by considering both cost and synchronization criteria. The total number of iterations is adjusted to 15, the swarm size is fixed to 30, and the algorithm is run 10 times by assigning different initial conditions independently. In addition, the lower and upper bounds for the coupling parameters *σ*_*c*1_ as well as *σ*_*c*2_ are restricted as *l*_*b*_ = [0 0] and *u*_*b*_ = [0.2 0.2].

When considering the standard deviation results *σ*_*eh*_, the cost function *C*_*cst*_ is minimized with the constraint that (*σ*_*eh*_ ≤ 0.001). In this case, the repeated running results are obtained as in Table 1. From Table 1, the mean optimal coupling parameters and the mean cost are calculated as (*σ*_*c*1_ = 0.00052, *σ*_*c*2_ = 0.00494) and *C*_*cst*_ = 1.8003, respectively. Under the same conditions, when the coupling parameter *σ*_*c*2_ = 0, the mean value of *σ*_*c*1_ and the cost *C*_*cst*_ are calculated as 0.08014 and 3.60782, respectively. Similarly, when the coupling parameter *σ*_*c*1_ = 0, the mean values of *σ*_*c*2_ and *C*_*cst*_ are respectively 0.00491 and 1.76668. Based on these results, it can be said that the cost of a first-order coupled network alone is greater than that of a high-order coupled network alone. However, the least cost is achieved in case of availability of the second-order couplings solely. On the other hand, when both couplings are active together, the cost decreases compared to the case with only first-order couplings. Therefore, the existence of *σ*_*c*2_ leads to a reduction in the cost of a first-order coupled network. From this point, it can be concluded that the high-order couplings have a positive effect on neural synchronization.

**Table 1:** The obtained optimal coupling parameters for the minimized cost function *C*_*cst*_ with the constraint (*σ*_*eh*_ ≤ 0.001) (*N*_*n*_ = 10)

	1	2	3	4	5	6	7	8	9	10	Mean
*(a)*											
*σ*_*c*1_	0.081	0.0795	0.0802	0.0802	0.0805	0.0805	0.083	0.0805	0.078	0.078	0.08014
*C*_*cst*_	3.6458	3.5797	3.6082	3.6082	3.623	3.623	3.7443	3.623	3.5115	3.5115	3.60782
*(b)*											
*σ*_*c*2_	0.0049	0.0049	0.0047	0.0050	0.0049	0.0051	0.0051	0.0047	0.0047	0.0051	0.00491
*C*_*cst*_	1.7769	1.7769	1.6951	1.8035	1.7485	1.8253	1.8253	1.695	1.695	1.8253	1.76668
*(c)*											
*σ*_*c*1_	0.000 01	0.000 01	0.000 95	0.000 95	0.000 78	0.000 77	0.000 28	0.000 28	0.000 86	0.000 32	0.000 52
*σ*_*c*2_	0.0053	0.0053	0.0049	0.0049	0.0049	0.0049	0.0047	0.0047	0.0049	0.0049	0.00494
*C*_*cst*_	1.9043	1.9043	1.8062	1.8062	1.7991	1.799	1.7037	1.7037	1.7879	1.7885	1.8003

### Effect of the fractional order of the neural network

**Fig. 10:**
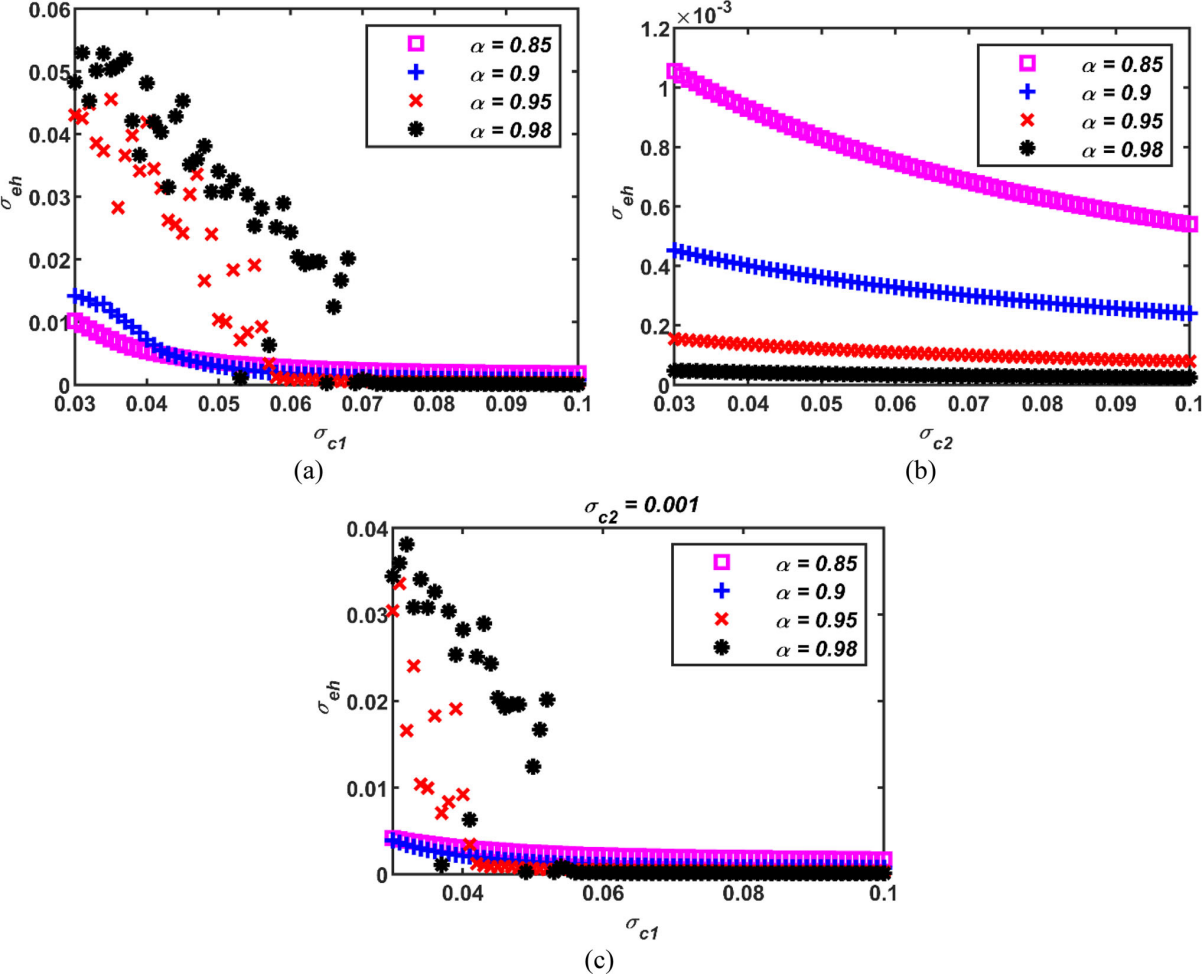
Standard deviation results of the neural network for different fractional orders (a) case with first order coupling only (b) case with second order coupling only and (c) case with first order as well as second order couplings; *σ*_*c*1_: first-order coupling strength, *σ*_*c*2_: second-order coupling strength, *σ*_*eh*_: standard deviations

To inspect the effect of fractional order parameter on neural synchronization, the variation of the performance parameter *σ*_*eh*_ with respect to coupling parameters *σ*_*c*1_ and *σ*_*c*2_ at different fractional orders is presented in Fig. 10. It is visible from Fig. 10a that the performance parameter *σ*_*eh*_ starts with a smaller value for the network with a lower fractional order and gradually decreases. On the contrary, for the network with a higher fractional order, the performance parameter *σ*_*eh*_ starts with a larger value and rapidly decreases up to an intersection point. Beyond this point, the network with a higher fractional order produces less error values and approaches a more synchronous state. Ultimately, compared to the network with a higher fractional order, the network with a lower fractional order requires a larger coupling parameter to produce less error. For instance, at *σ*_*c*1_ = 0.05, the standard deviation result of the neuronal network with the order of 0.85 is *σ*_*eh*_ = 0.0036, while the result of the neural network with the order of 0.95 is *σ*_*eh*_ = 0.010. Hence, the initial metric is smaller for the network with a lower order. However, at *σ*_*c*1_ = 0.1, the performance metric of the network with a fractional order of 0.85 is *σ*_*eh*_ = 0.0018, while the metric of the neural network with the order of 0.95 is *σ*_*eh*_ = 0.00034. Therefore, the network with a higher order produces less error in the final state. The reason for this is that a neuron model with smaller fractional order has a higher number of spikes in each burst, while a larger fractional order model has a higher frequency of neural response. So, a larger fractional order network can respond faster. On the other hand, it is clear from Fig. 10b that the network with a smaller fractional order starts with a larger error value compared to the network with a larger fractional order, and this trend continues throughout the entire variation range of *σ*_*c*2_. Therefore, it can be said that the network with a larger fractional order exhibits more synchronous behavior. Furthermore, it is apparent from Fig. 10a-b that the standard deviation results of only second-order coupled neuronal networks are at a lower level than those of only first-order coupled networks regardless of the fractional order parameter. For the case of Fig. 10c, where both second and first order couplings are active, the performance parameter starts with lower values but shows a behavior similar to the case where only first order coupling is active.

In conclusion, considering the performance parameter *σ*_*eh*_, decreasing the fractional order has not a positive contribution to neural synchronization. However, it can be said that as the fractional order increases, the neurons forming the network exhibit more similar behaviors.

### Effect of the size of the neural network

**Fig. 11:**
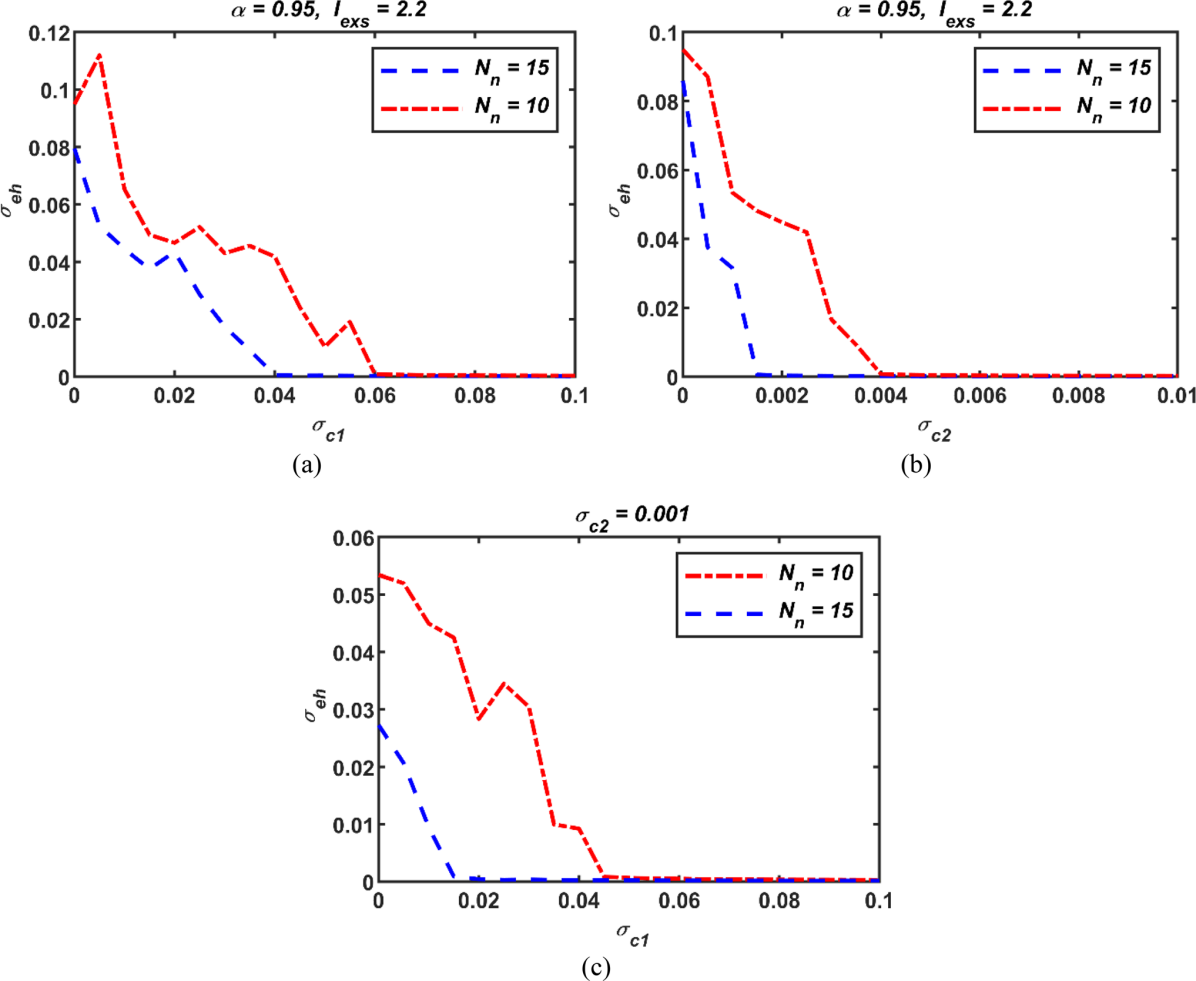
Standard deviation results of the neural network for different fractional orders (a) case with first order coupling only (b) case with second order coupling only and (c) case with first order as well as second order couplings; *σ*_*c*1_: first-order coupling strength, *σ*_*c*2_: second-order coupling strength, *σ*_*eh*_: standard deviations, *N*_*n*_: network size

To investigate the effect of network size on neural synchronization, the size of the network has been increased from *N*_*n*_ = 10 to 15. The results of the performance measure *σ*_*eh*_ obtained for the coupling parameters *σ*_*c*1_ and *σ*_*c*2_ are shown in Fig. 11. It is noticeable from Fig. 11 that increasing the size of the network reduces the values of performance parameter for all three cases. Therefore, neuronal synchronization can be achieved with smaller coupling parameter values. It should also be noted that the value of *σ*_*c*2_ is smaller compared to *σ*_*c*1_ at a certain value of the performance parameter.

On the other hand, a more accurate inference can be made by evaluating the cost function provided in (7) together with the increasing network size. For this reason, to determine the optimal coupling parameters in response to the increasing network size, the PSO algorithm has been utilized while keeping the set settings in Sect. "Coupling of the fractional-order neuronal models". Under these conditions, the calculated average values for the cases of only first-order coupling, second-order coupling solely, and both couplings being active are as follows: *i*) (*σ*_*c*1_ = 0.044, *C*_*cst*_ = 4.6236), *ii*) (*σ*_*c*2_ = 0.0017, *C*_*cst*_ = 2.2822) and *iii*) (*σ*_*c*1_ = 0.000338, *σ*_*c*2_ = 0.00167, *C*_*cst*_ = 2.3117). As can be seen from these results, the highest cost is the case with only first-order coupling. However, by incorporating second-order effects into the coupling mechanism, the cost is reduced and the synchronization is positively affected. In addition, the lowest cost is obtained only in the case of second-order coupling. The crucial point to note is that when the average coupling values obtained for *N*_*n*_ = 10 in Table 1 are compared with the values calculated for *N*_*n*_ = 15, it is clear that the average cost increases with increasing network size for all three coupling scenarios. Therefore, although the increased network size reduces the value of the coupling parameters for synchronization, it also increases the average cost and this aspect should be taken into account.

Considering the all-numerical simulation results, three main inferences can be made:

(i) Second-order interactions, when combined with first-order couplings, reduce both the cost of synchronization and the mean error values. Therefore, high-order interactions have a positive contribution to neural synchronization.
(ii) The fractional order of the HR neuron model affects the neural synchronization performance. As the fractional order parameter increases, the performance criterion *σ*_*eh*_ decreases. Because frequency of the neural response increases with the increasing fractional order, resulting in a faster response. Thus, a larger fractional order may be a better choice in terms of neural synchronization.
(iii) The network size also influences neural synchronization. Larger networks require smaller coupling parameters for the neural synchronization. However, it is crucial not to disregard the increased cost resulting from the increasing network size.

## Conclusion

In this study, high order interactions have been included in the coupling mechanism of the neural network consisting of fractional HR neuronal models. The effect of first- and second-order couplings on neural synchronization has been investigated individually and in combination. Numerical simulation results show that second-order couplings positively affect neural synchronization by reducing cost and pairwise coupling strength. Additionally, decreasing the fractional order has a negative effect on synchronization according to performance measures. On the other hand, the magnitude of coupling parameters necessary for neural synchronization decreases with increasing network size. Consequently, a neural network with high fractional order and both first- and high-order interactions active is more favorable for neural synchronization. In fractional neural networks, since all past values of each individual neuron are taken into account, as the network size increases, the calculation becomes quite difficult and the cost increases.

The effects of high-order interactions and fractional calculus on multilayer structures with map-based neuron models are possible research subjects in future studies. Because discrete map-based neuron models are computationally advantageous especially in case of increasing network size. On the other hand, the accuracy of the results in fractional calculus varies depending on the employed numerical method. If very sensitive methods are used, computational cost can be a serious problem. Another research topic may be synchronization status of fractional neural network in case of both high order interactions and energy exchange.

## Supplementary Information

Below is the link to the electronic supplementary material.

Supplementary file1 (DOCX 14 kb)

## Data Availability

Data sharing not applicable to this article as no datasets were generated or analyzed during the current study.
