# Supplementary material for: Effects of high-order interactions on synchronization of a fractional-order neural system
Source: Cogn Neurodyn. 2024 Jan 8;18(4):1877–93. doi: 10.1007/s11571-023-10055-z (PMC11639445; doi:10.1007/s11571-023-10055-z)
Supplement: Supplementary file 1 — Supplementary file1 (DOCX 14 kb) [file 11571_2023_10055_MOESM1_ESM.docx]

%%%%%%%%%%

%%%MAIN CODE

clearvars

% Sim time

TSim = 4000;

% time step:

h = 0.001;

% number of calculated mesh points:

n = round(TSim/h);

%fractional order

q = 0.98;

%define parameters of neuronal model d for bh, I for Iexs, xrest for xRs, mu for r

d = 3; I = 3.4; mu = 0.009; xrest = -1.6; s = 4, sigmac1 = 0; sigmac2 = 0;

% initial conditions of state variables

x0 = 1; y0 = 0; z0 = 0;

x(1) = 0; y(1) = 0; z(1) = 0;

% initial calculations

k1 = (h.^q)./q;

k2 = 1./gamma(q);

k3 = (h.^q)./gamma(q+2);

for i = 0:1:n

a(1) = i.^(q+1)-(i-q).*((i+1).^q);

for j = 0:1:i

%calculation of ai and bi coeffs

b(j+1) = k1.*((i-j+1).^q-(i-j).^q);

a(j+2) = (i-j+2).^(q+1)+(i-j).^(q+1)-2.*((i-j+1).^(q+1));

cx = y(j+1)-(x(j+1).^3)+d.*(x(j+1).^2)+I-z(j+1);

cy = 1-5.*(x(j+1).^2)-y(j+1);

cz = mu.*s.*(x(j+1)-xrest)-mu.*z(j+1);

sumbx(j+1) = b(j+1).*cx;

sumby(j+1) = b(j+1).*cy;

sumbz(j+1) = b(j+1).*cz;

sumax(j+1) = a(j+1).*cx;

sumay(j+1) = a(j+1).*cy;

sumaz(j+1) = a(j+1).*cz;

end

%prediction part

xp(i+1) = x0+k2.*sum(sumbx);

yp(i+1) = y0+k2.*sum(sumby);

zp(i+1) = z0+k2.*sum(sumbz);

%

%correction part

x(i+2) = x0+k3.*(yp(i+1)-xp(i+1).^3+d.*(xp(i+1).^2)+I-zp(i+1))+k3.*sum(sumax);

y(i+2) = y0+k3.*(1-5.*(xp(i+1).^2)-yp(i+1))+k3.*sum(sumay);

z(i+2) = z0+k3.*(mu.*s.*(xp(i+1)-xrest)-mu*zp(i+1))+k3.*sum(sumaz);

end

T = 0:h:TSim;

%plotting time series of state variables

plot(T,x); hold on;

plot(T,y); hold on;

plot(T,z);

%%%MAIN CODE END

%%%%%%%%%%%%%

%%%%%%%%

%FOR sigma1

%Set sigma1 and exclude from MAIN CODE

for i = 0:0.01:1

sigma2 = i;

%use respective parts from MAIN CODE HERE

end

%%%%%%%%

%%%%%%%%

%FOR sigma2

%Set sigma2 and exclude from MAIN CODE

for i = 0:0.01:1

sigma1 = i;

%use respective parts from MAIN CODE HERE

end

%%%%%%%%

%%%%%%%% OPTIMIZATION PART

%%%%%%

function pso_frak

%Define optimization options such as the maximum number of iterations and the swarm size

options = optimoptions('particleswarm','PlotFcns',@pswplotbestf,'MaxIter',50,'SwarmSize',50);

%define the lower bound on the search domain for sigmac1 and sigmac2

lb = [0.0000001 0.0000001];

%define the upper bound on the search domain for sigmac1 and sigmac2

ub = [2 2];

%call optimization function

[x,fval,exitflag,output] = particleswarm(@parameterfrac,2,lb,ub,options);

end

%%%%%%

%%%%%%

% optimization function

function yfin = parameterfrac(x)

% use x(1) and x(2) for sigma1 and sigma2, respectively;

%insert respective parts from MAIN CODE here

% insert check condition

end

%%%%%%

%%%%%%%% OPTIMIZATION PART END
